# Composition of sand fly fauna (Diptera: Psychodidae) and detection of *Leishmania* DNA (Kinetoplastida: Trypanosomatidae) in different ecotopes from a rural settlement in the central Amazon, Brazil

**DOI:** 10.1186/s13071-018-2743-6

**Published:** 2018-03-13

**Authors:** Erica Cristina da Silva Chagas, Arineia Soares Silva, Nelson Ferreira Fé, Lucas Silva Ferreira, Vanderson de Souza Sampaio, Wagner Cosme Morhy Terrazas, Jorge Augusto Oliveira Guerra, Rodrigo Augusto Ferreira de Souza, Henrique Silveira, Maria das Graças Vale Barbosa Guerra

**Affiliations:** 1 Universidade do Estado do Amazonas (Programa de Pós-graduação em Medicina Tropical/Programa de Pos-graduação em Clima e Ambiente), Manaus, Amazonas Brasil; 2 Fundação de Medicina Tropical Dr. Heitor Vieira Dourado, Manaus, Amazonas Brasil; 3 Fundação de Vigilância em Saúde do Estado do Amazonas, Manaus, Amazonas Brasil; 4 Instituto de Higiene e Medicina Tropical de Lisboa, Universidade Nova de Lisboa, Lisboa, Portugal

**Keywords:** Vectors of *Leishmania*, *Leishmania* DNA, Sand fly diversity and richness, Amazon region, Brazil

## Abstract

**Background:**

Phlebotomine sand flies (Diptera: Psychodidae) are vectors of *Leishmania* species, the etiological agents of leishmaniasis, which is one of the most important emerging infectious diseases in the Americas. In the state of Amazonas in Brazil, anthropogenic activities encourage the presence of these insects around rural homes. The present study aimed to describe the composition and distribution of sand fly species diversity among the ecotopes (intradomicile, peridomicile and forest) in an area of American cutaneous leishmaniasis transmission and detect natural infection with *Leishmania* DNA to evaluate which vectors are inside houses and whether the presence of possible vectors represents a hazard of transmission.

**Results:**

Phlebotomine sand flies were collected using light traps. A total of 2469 specimens representing 54 species, predominantly females (71.2%), were collected from four sites. Polymerase chain reaction analysis was performed on 670 samples to detect *Leishmania* DNA. Most of the samples (79.5%) were collected in the forest, with areas closer to rural dwellings yielding a greater abundance of suspected or proven vectors and a larger number of species containing *Leishmania* DNA. *Nyssomyia umbratilis* and *Bichromomyia flaviscutellata* were found near rural homes, and *Ny*. *umbratilis* was also found inside homes. *Leishmania* DNA was detected in different species of sand flies in all ecotopes, including species with no previous record of natural infection.

**Conclusions:**

There is no evidence that vectors of American cutaneous leishmaniasis are becoming established inside homes, but there are sand flies, including *Ny. umbratilis* and other possible vectors, in environments characterized by a human presence. These species continue to be predominant in the forest but are prevalent in areas closer to ecotopes with a greater human presence. The existence of proven or suspected vectors in this ecotope is due to the structural organization of rural settlements and may represent a hazard of transmission. Although the detection of *Leishmania* DNA in species that were not previously considered vectors does not mean that they are transmitting the parasite, it does show that the parasite is circulating in ecotopes where these species are found.

## Background

Phlebotomine sand flies (Diptera: Psychodidae) are important vectors of human diseases that have a great impact on public health, mainly as vectors of *Leishmania* species, the etiological agents of leishmaniasis [1]. This disease is endemic in 98 countries, posing a risk to an estimated 350 million people and infecting 0.7–1.2 million people annually [2–4]. Leishmaniasis is one of the most important emerging infectious diseases in the Americas [5].

There are approximately 1000 phlebotomine sand fly species, but only 98 species are proven or suspected vectors of human leishmaniasis. Phlebotomine sand flies in America include approximately 530 valid species, of which 135 species have been recorded in the state of Amazonas, Brazil [6–8]. The species of sand flies that have been described as the main vectors of American cutaneous leishmaniasis (ACL) in the Amazonas are *Nyssomyia umbratilis* and *Ny. anduzei*, which are vectors of *Leishmania* (*Viannia*) *guyanensis*, and *Bichromomyia flaviscutellata* and *Bi. olmeca nociva*, which are vectors of *L.* (*Leishmania*) *amazonensis* [9–11]. *L.* (*V.*) *guyanensis* is the most prevalent species in the region [12].

Previous studies have shown the presence of proven or putative vectors, mainly in the forest but also near houses. In the municipality of Manaus, the capital of Amazonas, in the western region of the Amazon basin, Feitosa & Castellón [13] observed the presence these insects also in the intradomicile and peridomicile areas of housing complexes near forest fragments. Moreover, Barbosa et al. [14] showed *Ny. umbratilis* could be observed in peridomicile areas of a rural community. Figueira et al. [15] collected most sand flies in peridomicile areas of a neighbourhood, a rural settlement, and two indigenous settlements in the Labrea municipality. Reis et al. [16] investigated natural *Leishmania* infection in sand flies collected in peridomiciles in a neighbourhood in Manaus near a forest reserve; however, the results were negative. These findings, in addition to the fact that some areas in Brazil show a change in ACL transmission patterns, such as the adaptation of some sand flies to the peridomiciliary environment [17], emphasize the importance of studies of the composition and distribution of sand flies in the Amazon region.

In Manaus, areas of ACL occurrence are associated with deforestation, the presence of houses on the outskirts of the forest and the presence of synanthropic animals near households. To address ACL, it is important to understand the composition and diversity of phlebotomine species, particularly in environments with greater anthropogenic pressure, such as the peridomicile and intradomicile. Thus, the present study aimed to describe the composition and distribution of sand fly species diversity among ecotopes (intradomicile, peridomicile and forest) in an area of ACL transmission, as well as to detect natural infection with *Leishmania* DNA, in order to evaluate which vectors are inside houses and whether they represent a hazard of transmission.

## Methods

### Study area

**Fig. 1:**
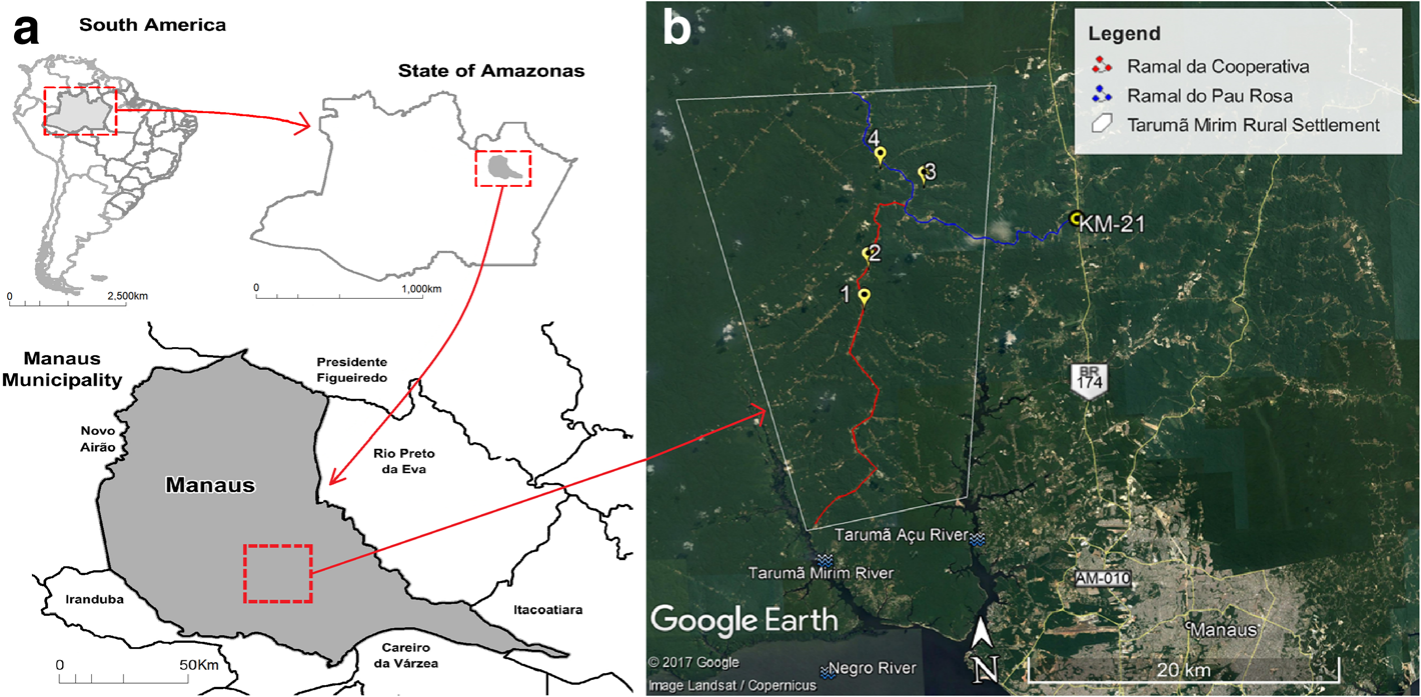
Map of the study area. **a** Location of collection area (Tarumã Mirim Rural Settlement) in Manaus Municipality, Amazonas State, Brazil. Source: CDC [48]. **b** Tarumã Mirim Rural Settlement with location of fixed collection points to sand fly sampling: Ramal da Cooperativa (red), points 1 and 2, and Ramal do Pau Rosa (blue), points 3 and 4. Source: Google Earth [49]

The study was conducted in a rural area of Manaus, northern Brazil, in the western Amazon (Fig. 1a). Manaus has an area of 11,401.092 km^2^ and an estimated 2,094,400 inhabitants [18]. In this region, leishmaniasis cases are concentrated in rural areas, such as the Tarumã Mirim Rural Settlement (2.792972°S, 60.036966°W), an important autochthonous transmission area. This settlement contains 42,910.76 hectares and is located northwest to Manaus, between the Tarumã Mirim and Tarumã Açu basins. The settlement is formed by two main branches, Ramal do Pau Rosa and Ramal da Cooperativa, from which numerous branch roads originate.

The predominant vegetation cover is ombrophile forest, with anthropogenic activities being quite intensive and including agricultural crops, coal production, livestock and secondary vegetation. The climate of the region is type "A" (Köppen classification), presenting two climatic variations: Af and Amw, hot and wet climates. The average annual temperature is 27 °C with a small thermal amplitude. The atmospheric humidity varies between 75–86%, and pluviometric indexes range between 1750–2500 mm a year [19, 20].

For the study, four fixed collection points were established along the two main branches (Fig. 1b), in dwellings where there was a record of at least one autochthonous case of ACL in the six months prior to the study.

### Phlebotomine sand fly collection and species identification

Phlebotomine sand flies were collected using 16 CDC (Centers for Disease Control and Prevention) light traps installed 1 m off the ground, for three consecutive days from 5:00 pm to 7:00 am, between May 2015 and April 2016, totalling 9216 hours of sampling effort. At each fixed point of collection, the traps were installed as follows: one intradomicile, one peridomicile and two in the forest (100 m and 400 m from the edge of the forest, denoted F100m and F400m, respectively). The distance from the edge of the forest to the dwellings varied greatly according to the fixed point of collection, ranging between 30–170 meters.

The collected males and body parts of the females (head and the last two abdominal segments) were mounted on glass slides with Berlese liquid and identified according to their external morphological characteristics, using the key developed by Young & Duncan [21] and Galati [22] and generic abbreviations proposed by Marcondes [23]. The thoraces and abdomen parts of the females were stored in 80% ethyl alcohol and kept at -20 °C for subsequent molecular analysis to detect *Leishmania* DNA.

### Detection of *Leishmania* DNA

To detect *Leishmania* DNA in phlebotomine sand flies, only non-engorged and egg-free females were used, in order to minimize the detection of the parasite in undigested blood [24]. Females were individually processed or grouped into pools of up to 10 specimens, organized according to species, ecotope, collection point and date. A total of 670 samples were formed for molecular analysis, including individual and pooled specimens.

DNA extraction was performed in pools of 5–10 specimens, using the Purelink™ Genomic DNA Mini Kit (Invitrogen, Carlsbad, USA), following the manufacturer's instructions. However, for individual phlebotomine sand flies and pools of 2–4 specimens, DNA extraction was done with Chelex® 100 resin (BioRad, Hercules, USA) because through this technique, it is possible to extract a greater amount of DNA. During the process of DNA extraction, every 10 samples included a blank to confirm that the samples were not contaminated.

*Leishmania* DNA was detected using multiplex polymerase chain reaction (PCR), using as target DNA: (i) a mini-circle of kDNA (116 bp) for identification of the genus *Leishmania*, using the primers 13a (5'-GTG GGG GAG GGG CGT TCT-3') and 13b (5'-ATT TTA CAC CAA CCC CCA GTT-3') [25]; and (ii) a fragment of the *cox*1 gene (658 bp) to identify DNA from sand flies and confirm the quality of DNA extraction, using the primers LCO1490 (5'-GGT CAA CAA ATC ATA AAG ATA TTG G-3') and HCO2198 (5'-TAA ACT TCA GGG TGA CCA AAA AAT CA-3') [26]. The reaction mixture contained 5 μL of extracted DNA, 0.15 μL of each primer (0.4 μM), 0.75 μL of MgCl_2_ (3.5 μM), 0.5 μL of dNTPs (0.2 mM), 1 U Taq polymerase and Buffer 1×, for a total volume of 25 μl. The multiplex reaction was performed with an initial denaturation of 94 °C for 4 min, followed by 10 cycles of 94 °C, 60 °C, and 72 °C, 1 min each and 20 cycles of 94 °C, 50 °C, and 72 °C, 1 min each. The final extension was performed at 72 °C for 5 min. In each reaction, a no-template control was included, as well as positive controls for phlebotomine and *Leishmania* reference strain DNA.

The reaction products were subjected to 2% agarose gel electrophoresis in 1× TBE, stained with ethidium bromide. The approximate size of the amplification product, in terms of base pairs, was determined by comparison with a 100 bp and 50 bp marker. The amplified products were visualized under ultraviolet light and subsequently photographed.

### Statistical analysis

Quantification of phlebotomine sand flies collected in the study area was expressed through absolute abundance, relative abundance and standardized index of species abundance. The Standardized Index of Species Abundance (SISA) was calculated as described by Roberts & Hsi [27]. SISA values range from 0 to 1, and species abundance was considered high when the SISA value was close to the maximum value of 1. Species diversity was assessed using the Shannon’s Index (H′), calculated with Diversidade de Espécies (DivEs) software version 4.0 (http://dives.ebras.bio.br/guide_dives.aspx). The distribution of species in the study period was calculated by the Constancy Index according to Silveira Neto et al. [28], which classifies species as constants (when present in more than 50% of collections), accessory (present in 25–50% of collections), or accidental (present in less than 25% of collections). Abundance and Shannon’s index values were compared between ecotopes using the Student's t-test to determine significance, performed with Stata software v. 13. The minimum infection rate was calculated with the following formula: number of positive pools × 100 / total number of females in pool [29, 30]. Maps were generated using the software MapInfo Pro^TM^ v. 16.

## Results

A total of 2469 phlebotomine sand flies were collected, representing 54 species and 13 genera: *Psychodopygus* (*Ps.*, 10 spp.), *Psathyromyia* (*Pa.*, 9 spp.), *Evandromyia* (*Ev.*, 8 spp.), *Lutzomyia* (*Lu.*, 4 spp.), *Micropygomyia* (*Mi.*, 3 spp.), *Nyssomyia* (*Ny.*, 3 spp.), *Sciopemyia* (*Sc.*, 3 spp.), *Trichopygomyia* (*Ty.*, 3 spp.), *Bichromomyia* (*Bi.*, 2 spp.), *Migonemyia* (*Mg.*, 2 spp.), *Pressatia* (*Pr.*, 2 spp.), *Trichophoromyia* (*Th.*, 2 spp.), *Viannamyia* (*Vi.*, 2 spp.), and *Pintomyia* (*Pi.*, 1 sp.) (Table 1).

**Table 1:** Species of sand flies from Tarumã Mirim Rural Settlement, Manaus Municipality, Amazonas State, Brazil, collected with CDC light traps from May 2015 to April 2016

Species [Reference]	Id		Pd		F100m		F400m		Total	%	Ratio M/F	SISA	Rank
	M	F	M	F	M	F	M	F					
*Ty. trichopyga* Floch & Abonnenc, 1945	10	2	8	4	28	48	209	183	492	19.9	1.08	0.9	4
*Mi. rorotaensis* Floch & Abbonenc, 1944	5	53	3	21	27	185	23	85	402	16.3	0.17	1.0	1
*Ny. umbratilis* Ward & Fraiha, 1977^a^ [2, 40, 41]	4	16	28	13	45	112	54	49	321	13.0	0.69	1.0	2
*Sc. sordellii* Shannon & Del Ponte, 1927	8	94	3	60	12	22	3	34	236	9.6	0.12	0.9	3
*Ny. anduzei* Rozeboom, 1942^a^ [2, 41]	1	8	–	4	4	89	10	49	165	6.7	0.10	0.9	5
*Ps. davisi* Root, 1934^a^ [42, 43]	–	1	1	7	12	75	4	16	116	4.7	0.17	0.8	9
*Ev. sericea* Floch & Abbonenc, 1944	1	4	16	18	7	5	1	9	61	2.5	0.69	0.8	8
*Sc. nematoducta* Young & Arias, 1984	3	5	5	3	7	7	10	12	52	2.1	0.93	0.8	6
*Ev. georgii* Freitas & Barrete, 2002	–	3	–	9	–	16	–	22	50	2.0	–	0.8	7
*Ps. geniculatus* Mangabeira, 1941	–	–	5	1	2	32	4	1	45	1.8	0.32	0.5	19
*Bi. olmeca nociva* Young & Arias, 1982^a^ [2, 41]	–	1	–	1	–	8	1	30	41	1.7	0.03	0.6	18
*Pr. triacantha* Mangabeira, 1942	–	4	1	2	1	7	8	17	40	1.6	0.33	0.7	10
*Ps. sq. squamiventris* Lutz & Neiva, 1912^a^ [2]	–	1	1	–	6	7	2	21	38	1.5	0.31	0.6	13
*Mi. micropyga* Mangabeira, 1942	–	1	–	1	–	21	–	13	36	1.5	–	0.6	15
*Th. eurypyga* Martins, Falcão & Silva, 1963	–	1	2	–	1	6	10	7	27	1.1	0.93	0.6	17
*Ev. monstruosa* Floch & Abonnenc, 1944	–	1	–	4	2	8	–	11	26	1.1	0.08	0.7	11
*Pa. dendrophyla* Mangabeira, 1942	–	1	10	–	2	7	2	3	25	1.0	1.27	0.6	12
*Pr. trispinosa* Mangabeira, 1942	–	–	1	–	1	–	22	–	24	1.0	–	0.3	27
*Ps. corossoniensis* Le Pont & Pajot, 1978	–	2	–	–	–	16	–	4	22	0.9	–	0.5	20
*Bi. flaviscutellata* Mangabeira, 1942^a^ [2, 40, 41]	–	–	–	2	2	12	1	5	22	0.9	0.16	0.5	21
*Pa. aragaoi* Costa Lima, 1932	–	–	2	–	4	5	7	4	22	0.9	1.44	0.5	22
*Ny. antunesi* Coutinho, 1939^a^ [2, 40]	–	3	–	7	–	6	–	5	21	0.9	–	0.6	14
*Th. ruii* Arias & Young, 1982	1	–	–	2	11	–	5	2	21	0.9	4.25	0.6	16
*Vi. tuberculata* Floch e Abonnenc, 1941	–	–	–	–	–	9	–	10	19	0.8	–	0.3	29
*Lu. evangelistai* Martins & Fraiha, 1971	–	1	–	–	–	7	–	7	15	0.6	–	0.4	25
*Vi. furcata* Mangabeira, 1941	1	–	–	1	1	7	1	3	14	0.6	0.27	0.5	23
*Ps. paraensis* Costa Lima, 1941^a^ [2, 40]	–	–	1	–	–	7	–	6	14	0.6	0.08	0.4	26
*Mi. pilosa* Damasceno & Causey, 1944	–	1	–	1	–	3	–	4	9	0.4	–	0.4	24
*Pa. shannoni* Dyar, 1929	–	–	1	1	2	2	2	–	8	0.3	1.67	0.3	30
*Lu. gomezi* Nitzulescu, 1931^a^ [2, 40]	–	1	–	–	2	3	2	–	8	0.3	1.00	0.3	32
*Ps. hi. hirsutus* Mangabeira, 1942	–	–	–	1	1	5	–	1	8	0.3	0.14	0.2	37
*Ps. ayrozai* Barretto & coutinho, 1942^a^ [2, 40, 41]	–	–	–	1	3	1	1	1	7	0.3	1.33	0.2	33
*Ps. claustrei* Abonnenc, Léger & Fauram, 1979	–	–	–	1	–	4	1	1	7	0.3	0.17	0.2	34
*Pa. dreisbachi* Causey & Damasceno, 1945	1	2	–	–	–	1	–	2	6	0.2	0.20	0.3	31
*Ps. chagasi* Costa Lima, 1941	–	–	–	–	–	2	–	4	6	0.2	–	0.2	40
*Lu. falcata* Young, Morales & Ferro, 1994	–	1	–	1	–	1	–	2	5	0.2	–	0.3	28
*Ty. ratcliffei* Arias, Ready & Freitas, 1983	1	–	–	–	–	–	3	–	4	0.2	–	0.2	36
*Ps. amazonensis* Root, 1934	–	–	1	–	–	1	1	1	4	0.2	1.00	0.2	38
*Ty. longispina* Mangabeira, 1943	–	–	–	–	–	–	2	2	4	0.2	1.00	0.1	44
*Pa. inflata* Floch & Abonnenc, 1944	1	–	–	1	–	–	1	–	3	0.1	2.00	0.2	35
*Mg. migonei* França, 1920^a^ [2, 40, 41]	–	–	–	–	–	1	1	1	3	0.1	0.50	0.1	45
*Pa. lutziana* Costa Lima, 1932	–	1	–	–	1	–	–	–	2	0.1	1.00	0.2	39
*Pa. runoides* Fairchild & Hertig, 1953	–	–	2	–	–	–	–	–	2	0.1	–	0.2	41
*Pa. barrettoi barrettoi* Mangabeira, 1942	–	–	1	–	–	–	1	–	2	0.1	–	0.1	43
*Pi. damascenoi* Mangabeira, 1941	–	–	–	–	–	–	–	2	2	0.1	–	0.1	48
*Ev. infraspinosa* Mangabeira, 1941	–	–	–	–	–	1	–	1	2	0.1	–	0.1	49
*Pa. scaffi* Damasceno & Arouck, 1956	–	–	–	–	–	1	1	–	2	0.1	1.00	0.1	50
*Ev. williamsi* Damasceno, Causey & Arouck, 1945	–	–	–	–	1	–	–	1	2	0.1	1.00	0.1	51
*Mg. moucheti* Pajot & Le Pont, 1978	1	–	–	–	–	–	–	–	1	0.0	–	0.1	42
*Ev. pinottii* Damasceno & Arouck, 1956	–	–	1	–	–	–	–	–	1	–	–	0.1	46
*Evandromyia* sp. de Baduel Floch & Abonnenc, 1945	–	–	1	–	–	–	–	–	1	–	–	0.1	47
*Ev. inpai* Young & Arias, 1977	–	–	–	–	–	–	–	1	1	–	–	0.0	52
*Sc. preclara* Young & Arias, 1984	–	–	–	–	–	–	–	1	1	–	–	0.0	53
*Lu. spathotrichia* Martins, Falcão & Silva, 1963	–	–	–	–	–	–	–	1	1	–	–	0.0	54
Total	38	208	94	167	185	750	393	634	2,469	100	0.40	–	–

*Abbreviations*: *Id* intradomicile, *Pd* peridomicile, *F100m* forest 100 m from edge, *F400m* forest 400 m from edge, *M* male, *F* female, *SISA* Standardized Index of Species Abundance

^a^Species that are suspected or proven vectors

The most abundant species were *Ty. trichopyga* (19.9%; 4th rank SISA), *Mi. rorotaensis* (16.3%; 1st rank SISA), *Ny. umbratilis* (13%; 2nd rank SISA), *Sc. sordellii* (9.6%; 3rd rank SISA), *Ny. anduzei* (6.7%; 5th rank SISA) and *Ps. davisi* (4.7%; 9th rank SISA), which together comprised 70.1% of the total specimens captured (Table 1). The male/female ratio was 0.40, with 71.2% females and 28.8% males.

The greatest abundance and species richness was observed in the forest environment. In the F400m area, 1027 (41.6%) specimens and 49 species were collected. The predominant species were *Ty. trichopyga* (392 specimens, 38.2%), *Mi. rorotaensis* (108 specimens, 10.5%) and *Ny. umbratilis* (103 specimens, 10%). In the F100m area, 935 (37.9%) specimens and 42 species were collected, with a predominance of *Mi. rorotaensis* (212 specimens, 22.7%), followed by *Ny. umbratilis* (157 specimens, 16.8%) and *Ny. anduzei* (93 specimens, 9.9%).

The peridomicile showed 261 (10.6%) specimens and 36 species. The most abundant species was *Sc. sordellii* (63 specimens, 24.1%), followed by *Ny. umbratilis* (41 specimens, 15.7%) and *Ev. sericea* (34 specimens, 13%). In the intradomicile area, 246 (10%) specimens and 29 species were collected. *Sciopemyia sordellii* (102 specimens, 41.5%) was predominant, followed by *Mi. rorotaensis* (58 specimens, 23.6%) and *Ny. umbratilis* (20 specimens, 8.1%) (Table 1).

**Fig. 2:**
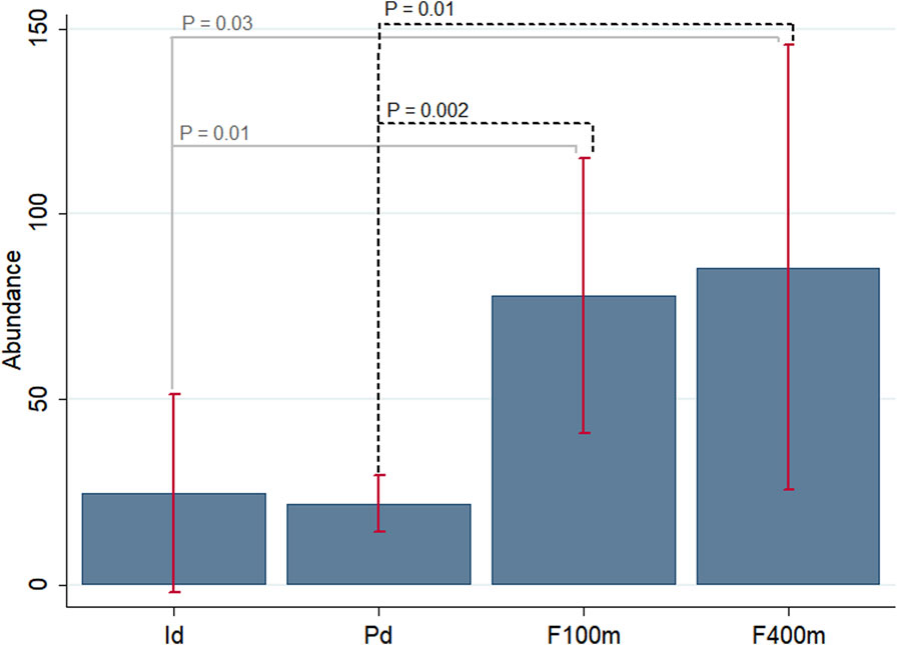
Abundance of phlebotomine sand flies. Phlebotomine sand flies from Tarumã Mirim Rural Settlement, Manaus Municipality, Amazonas State, Brazil, collected May 2015 to April 2016, at the following sampling sites: intradomicile (Id), peridomicile (Pd), 100 m from the edge of the forest (F100m), and 400 m from the edge of the forest (F400m)

A comparison of the mean phlebotomine sand fly abundance during the total collection time revealed a statistical difference between intradomicile and F100m (*t*_(20)_ = -2.49, *P* = 0.01); intradomicile and F400m (*t*_(20)_ = -1.92, *P* = 0.03); peridomicile and F100m (*t*_(22)_ = -3.26, *P* = 0.002); and peridomicile and F400m (*t*_(22)_ = -2.33, *P* = 0.01) (Fig. 2).

**Fig. 3:**
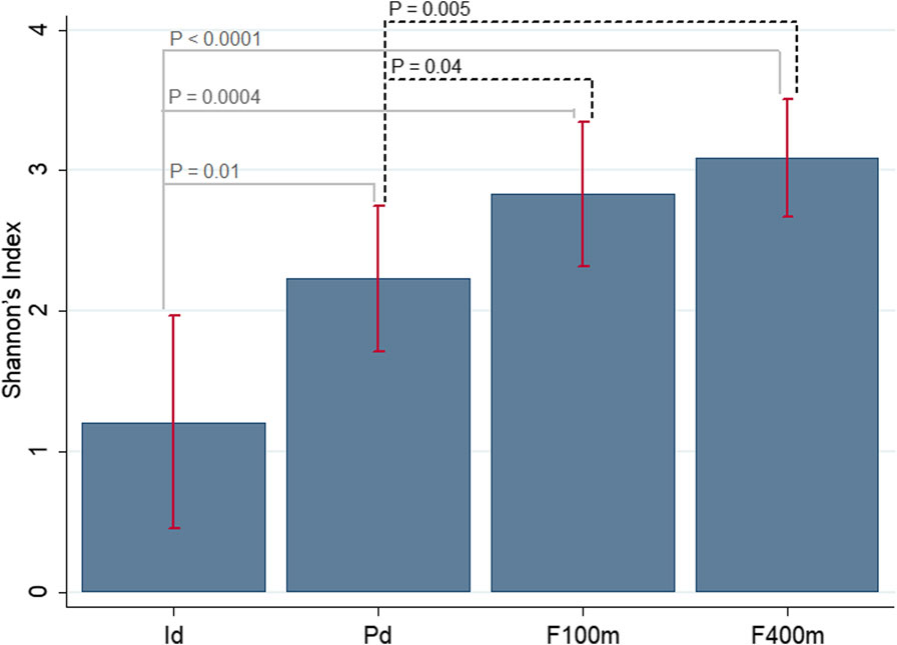
Shannon’s Index for phlebotomine sand flies. Phlebotomine sand flies from Tarumã Mirim Rural Settlement, Manaus Municipality, Amazonas State, Brazil, collected May 2015 to April 2016, at the following sampling sites: intradomicile (Id), peridomicile (Pd), 100 m from the edge of the forest (F100m) and 400 m from the edge of the forest (F400m)

The ecotope that had the highest diversity was F100m (H' = 3.93), followed by the peridomicile (H′ = 3.81). The comparison of the average diversity for total collection time showed a statistical difference between intradomicile and peridomicile (*t*_(22)_ = -2.46, *P* = 0.01); intradomicile and F100m (*t*_(22)_ = -3.90, *P* = 0.0004); intradomicile and F400m (*t*_(22)_ *=* -4.78, *P* < 0.0001); peridomicile and F100m (*t*_(22)_ = -1.81, *P* = 0.04); and peridomicile and F400m (*t*_(22)_ = -2.83, *P* = 0.005) (Fig. 3).

Among the species suspected or proven to be vectors, *Bi. olmeca nociva*, *Ny. anduzei*, *Ny. antunesi*, *Ny. umbratilis*, *Ps. davisi* and *Ps. sq. squamiventris* were found in all evaluated ecotopes. *Bichromomyia flaviscutellata*, *Ps. ayrozai* and *Ps. paraensis* were found in the peridomicile and in the forest. *Lutzomyia gomezi* was present in the intradomicile and in the forest, and *Mg. migonei* was found exclusively in the forest. *Bichromomyia olmeca nociva*, *Ny. anduzei*, *Ny. umbratilis* and *Ps. davisi* were classified using the constancy index as constant species throughout the study period (Table 2).

**Table 2:** Constancy index for species suspected or proven to be vectors from Tarumã Mirim Rural Settlement, Manaus Municipality, Amazonas State, Brazil, collected with CDC light traps from May 2015 to April 2016

Suspected or proven vectors	Constancy index	Status
*Bichromomyia flaviscutellata*	30.6	Accessory
*Bichromomyia olmeca nociva*	52.8	Constant
*Lutzomyia gomezi*	13.9	Accidental
*Migonemyia migonei*	8.3	Accidental
*Nyssomyia anduzei*	66.7	Constant
*Nyssomyia antunesi*	27.8	Accessory
*Nyssomyia umbratilis*	91.7	Constant
*Psychodopygus ayrozai*	16.7	Accidental
*Psychodopygus davisi*	69.4	Constant
*Psychododpygus paraensis*	22.2	Accidental
*Psychodopygus sq. squamiventris*	50.0	Accessory

Various species of phlebotomine sand flies tested positive for *Leishmania* DNA in all ecotopes evaluated (Table 3). Of the 670 analysed samples, 54 (8.1%) samples belonging to 21 phlebotomine species were positive for the presence of *Leishmania* DNA. *Nyssomyia umbratilis* and *Ny. anduzei* had minimum infection rates of 2.7% and 6.2%, respectively.

**Table 3:** Distribution of positive sand fly samples for *Leishmania* DNA, from Tarumã Mirim Rural Settlement, Manaus Municipality, Amazonas State, Brazil, collected with CDC light traps from May 2015 to April 2016, according to ecotope

Species	Positive pools by ecotope				Total of pools by examined	Minimum infection rate
	Id	Pd	F100m	F400m		
*Bichromomyia flaviscutellata*	–	1	1	1	11	16.7
*Bichromomyia olmeca nociva*	–	–	1	2	23	7.5
*Evandromyia monstruosa*	–	–	2	–	20	8.3
*Evandromyia sericea*	–	1	–	–	21	2.8
*Lutzomyia gomezi*	–	–	1	–	4	25.0
*Nyssomyia anduzei*	–	–	6	3	43	6.2
*Nyssomyia antunesi*	1	–	–	–	13	5.0
*Nyssomyia umbratilis*	–	–	2	3	60	2.7
*Psathyromyia aragaoi*	–	–	1	–	6	11.1
*Psathyromyia dreisbachi*	–	–	1	–	5	20.0
*Psathyromyia lutziana*	1	–	–	–	1	100
*Psychodopygus amazonensis*	–	–	1	1	2	100
*Psychodopygus ayrozai*	–	–	1	1	3	66.7
*Psychodopygus chagasi*	–	–	1	1	6	33.3
*Psychodopygus claustrei*	–	–	1	–	4	16.7
*Psychodopygus corossoniensis*	–	–	3	3	10	27.3
*Psychodopygus davisi*	–	–	1	3	33	4.2
*Psychodopygus hirsutus hirsutus*	–	1	1	1	3	42.9
*Psychodopygus sq. squamiventris*	–	–	–	2	19	7.1
*Sciopemyia sordellii*	1	1	–	1	63	1.4
*Trichophoromyia eurypyga*	–	–	1	–	14	7.1

*Abbreviations*: *Id* intradomicile, *Pd* peridomicile, *F100m* forest 100 m from edge, *F400m* forest 400 m from edge

## Discussion

Our samples included 54 species of phlebotomine sand fly among the 67 species registered in Manaus and nearby municipalities [17]. These species were distributed between the intradomicile, peridomicile and forest. The majority of sand fly fauna was captured in forest environments, which was expected because the species prefer a primarily wild habit. Other studies carried out in different areas of Manaus forests also found high species richness, such as those by Arias & Freitas [31], Castellón et al. [32], Dias-Lima [33], Barbosa et al. [14] and Gomes et al. [34], who found 50, 57, 39, 49 and 58 species of sand fly, respectively. Our findings showed a high species richness of phlebotomine sand flies in the Tarumã Mirim Rural Settlement. Located in the northwest of Manaus, this settlement is an important autochthonous transmission ACL area.

The most abundant species found in the study were *Ty. trichopyga*, *Mi. rorotaensis*, *Ny. umbratilis*, *Sc. sordellii*, *Ny. anduzei* and *Ps. davisi*. Of these species, *Ny. umbratilis*, *Ny. anduzei* and *Ps. davisi* have already been associated with ACL transmission. In the Amazonas State, four species have been confirmed as vectors of ACL: *Ny. umbratilis*, *Ny. anduzei*, *Bi. flaviscutellata* and *Bi. olmeca nociva* [14, 31, 35]. These species of sand flies were found in this study and tested positive for *Leishmania* DNA. *Nyssomyia umbratilis* and *Ny. anduzei* had higher abundances and minimum infection rates at 2.7% and 6.2%, respectively. These findings are in agreement with the observed epidemiological situation, as *L.* (*V.*) *guyanensis* is the predominant species both in the state of Amazonas and in Manaus [5, 12, 36].

*Nyssomyia umbratilis*, the primary vector of *L.* (*V.*) *guyanensis*, is the main etiologic agent of ACL in Amazon and is associated with both cutaneous and mucosal forms of the disease [12, 15, 37]. This species is found primarily in forest environments but previously was recorded in intradomicile locations in the Amazonas state in only recently inhabited areas near residual forest [13], perhaps because the collection methods used by many surveys do not include the intradomicile. The presence of *Ny. umbratilis* in the peridomicile was reported in: (i) Manaus, in a rural community (24 specimens) [38], and an area near residual forest (23 specimens) [16]; (ii) Presidente Figueiredo, in a rural settlement (38 specimens) [17]; and (iii) Tabatinga, in rural settlements (2 specimens) [39]. The collection methods adopted in our study made it possible to observe the presence of *Ny. umbratilis* not only in the peridomicile but also in the intradomicile, with a total of 61 specimens observed in these ecotopes.

According to Rangel & Lainson [40], in areas with a high concentration of ACL, it is mistakenly believed that *Ny. umbratilis* is adapting to the peridomicile. In the present study, the highest abundance was observed in the forest (F100m location), showing that this species may be approaching environments with a greater human presence. Specimens with *Leishmania* DNA, however, were collected only in the forest, thereby suggesting the possibility of transmission in this area by this vector only in forest environments.

Species related to *Leishmania* spp. transmission, *Ny. umbratilis* [*L.* (*V*.) *guyanensis*], *Ny. anduzei* [*L.* (*V.*) *guyanensis*] and *Ps. davisi* [*L.* (*V.*) *naiffi*] [2, 41–43], presented high SISA values of 1.0, 0.9 and 0.8, respectively. In addition, the fact that these species were considered constant according to the constancy index highlights the potential for transmission and partially explains why this disease is endemic to the area, contributing to the higher transmission levels found in Manaus. However, the constancy index has limitations because of the use of a single method of collection; other species that, owing to their biology, are not attracted in abundance by CDC may exhibit errors in classification through the use of this index.

Specimens of *Bi. flaviscutellata* and *Ny. antunesi* in the peridomicile and intradomicile, respectively, were found to contain *Leishmania* DNA. *Bichromomyia flaviscutellata* is a vector of *L.* (*L*.) *amazonensis*, causing cutaneous leishmaniasis, mucosal leishmaniasis and diffuse cutaneous leishmaniasis in the Amazon [43]. In a study carried out in a campinarana area in Manaus, it was the species with the highest density [44]. Our data showed low abundance of this vector (22 specimens), with only two specimens observed in the peridomicile, thus reducing the chance of human-vector contact and transmission. This species is usually found near the ground biting small rodents, but in areas where it occurs at greater abundance, it can bite humans [45].

*Nyssomyia antunesi* is a suspected vector of *L.* (*V*.) *lindenbergi* and of *Leishmania* spp. in Pará and in some areas of the Colombian Amazon Forest, respectively. In Colombia, this species has developed intradomiciliary habits [15, 43]. *Nyssomyia antunesi* is also suspected to be a vector of *Leishmania* in the municipality of Labrea, Amazonas, where it was found in high abundance in intra- and peridomiciliary locations [15]. Ramos et al. [17] observed that *Ny. antunesi* was the most abundant species under similar environmental conditions of a rural settlement, and it was more abundant in the peridomicile. In this study, although this species was found at low abundance in the intradomicile, *Leishmania* DNA was observed in sand flies collected in this environment, thereby representing a risk of leishmaniasis transmission due to the highly anthropophilic nature of this insect.

Other species that are suspected or proven vectors of *Leishmania* spp. were observed in this study but are not important transmitters of ACL in Amazonas. Their presence indicates the potential receptivity of the area to other *Leishmania* spp. Specimens of *Ps. ayrozai*, *Ps. sq. squamiventris*, *Lu. gomezi* and *Ny. antunesi* were found to be positive for *Leishmania* DNA. Other species contained *Leishmania* DNA and were not related to ACL transmission. While this finding does not mean that these species are transmitting parasites, it does show that parasites are circulating in ecotopes where these species are found.

Among the evaluated ecotypes, differences in species composition and abundance were observed. In the F400m location there was an elevated abundance of phlebotomine species. However, the F100m areas showed a greater abundance of species that have already been implicated in the transmission of ACL (confirmed or suspected) and more specimens positive for the presence of *Leishmania* DNA. A significant difference was not observed in the abundance and diversity of sand flies among these ecotopes, both are forest environments.

The intra- and peridomicile were not significantly different in terms of abundance but differed in terms of diversity indexes. The intradomicile showed a low value for the Shannon’s index (H′) due the predominance of *Sc. sordellii* (41.5%) followed by *Mi. rorotaensis* (23.6%).

The proximity of forest ecotopes to dwellings where there is a considerable human presence can attract sand flies to peridomicile and intradomicile locations. Phlebotomine sand flies are capable of flying short distances but are attracted by light and seek the blood of humans and synanthropic animals, increasing the probability of chance encounters between humans and vectors.

There have been few studies on natural infection by *Leishmania* in the Amazonas state, and most involved the dissection of individual sand flies to detect the presence of trypanosomatid forms [10, 11, 16, 39, 46, 47]. The detection of *Leishmania* using molecular methods such as PCR has been performed in only two other studies of the Amazonas [7, 9]. Silva et al. [9] recorded the presence of *Leishmania* DNA in *Th. ubiquitalis*, *Ev. apurinan*, *Ny. umbratilis*, *Ny. yuilli yuilli*, *Ps. davisi* and *Sc. servulolimai* in an indigenous settlement in the Labrea municipality. Pereira Júnior et al. [7] observed the presence of *Leishmania* DNA in *Th. ubiquitalis* and *Ps. davisi* in terra firme and varzea environments in the Tefé municipality.

In Manaus, information on natural infection by *Leishmania* is scarce, despite its epidemiological importance for ACL in the Amazonas, which is one of the main areas where this disease is endemic. Arias & Freitas [10], Arias et al. [11] and Pinheiro et al. [47] detected the presence of *Leishmania* DNA in *Ny. umbratilis*, *Ny. anduzei*, *Bi. flaviscutellata* and *Bi. olmeca nociva*, using the dissection method. This lack of data highlights the relevance of the present study and demonstrates that, until now, there has been no evidence that ACL vectors are becoming increasingly associated with human residences.

While there are more sand flies such as *Ny. umbratilis* and other possible vectors in the forest, they were observed closer to environments with a greater human presence, showing that this species may be approaching these environments. In the Tarumã Mirim Rural Settlement, there are about 1,600 residents, with about 70% of the adults and children exhibiting a history of leishmaniasis (Barbosa, unpublished data). As this area is near the urban area in Manaus and reasonably accessible, there is a large flow of people visiting the settlement on weekends, holidays and during vacation periods. The presence of potential vector species in intradomicile and peridomicile locations, and of *Leishmania* DNA in species circulating in different environments, contributes to an increased hazard of ACL transmission in this area.

## Conclusions

There is a high level of species diversity of sand flies in the Tarumã Mirim Rural Settlement. Sand fly species vary across ecotopes and are more abundant in forest ecotopes than in peridomicile ecotopes. Some important species implicated as vectors were found in intradomicile and peridomicile locations due to the structural organization of rural settlements and contributing significantly to an increased hazard of ACL transmission. The detection of *Leishmania* DNA in species not considered vectors does not mean that these species are transmitting the parasite, but rather that the parasite is circulating in environments where these specimens were found.

## Abbreviations

ACL: American cutaneous leishmaniasis
CDC: Centers for Disease Control and Prevention
DNA: Deoxyribonucleic acid
F100m: Forest 100 m area
F400m: Forest 400 m area
H′: Shannon’s index
kDNA: Kinetoplastid DNA
PCR: Polymerase chain reaction
SISA: Standardized Index of Species Abundance
